# The Climate Crisis and Other Animals

**DOI:** 10.1017/awf.2025.13

**Published:** 2025-03-14

**Authors:** Dorien Braam

**Affiliations:** London School of Hygiene and Tropical Medicine

This timely monograph provides an extensive review of the animal-industrial complex analysing its role in the climate crisis. Reflected in the second part of the title ‘*and other animals*’, the author argues that climate change is currently largely considered as an existential crisis for humanity, while the impact on other animals and non-human species remains largely ignored. With the aim to ‘*consider human-animal relations in the emergences and effects of the climate crisis and to explore the prospects and strategies for real transformatory change*’, the book contributes to interdisciplinary climate and animal welfare studies by bridging theory and practice in advocating for advanced intersectional approaches to counter the animal-industrial complex as one approach to mitigate climate change.

The book is divided into two distinct parts, starting with an elaboration on how the climate crisis is largely a ‘*crisis of anthropocentrism and human exceptionalism*’, rooted in political and socio-economic factors. The author argues that the use of the human-centred term ‘*Anthropocene*’ risks depoliticising the climate crisis, promoting the use of ‘*Capitalocene*’ to uncover the uneven distribution of the costs and benefits of climate change. Capitalocene allows for a more comprehensive consideration of capitalist, colonial, and patriarchal developments which have led to the climate crisis, while unravelling systems of power, profit, and production, such as the reliance on multispecies exploitation.

The author subsequently argues that social sciences through Critical Animal Studies (CAS) can play an important role in exploring how animal exploitation intersects with class, gender, and racialised relations, in itself contributing to the climate crisis. The book thus brings a novel perspective by discussing how CAS can unpack the ways intersectional capitalist, gendered, and colonial discourses have led to the current crisis. This involves addressing the human-nature dichotomy that has led to oppression and the externalisation of emissions from costs of production, reflected in previous published literature discussing the ‘true’ cost of labour and food. The potential of CAS is described through its framing of capitalism in reducing value of the more-than-human, animalisation and intersecting forms of exploitation, with agricultural exploitation of non-human animals significantly contributing to climate change in particular.

Introducing concepts from a host of seminal works, the author describes how violence against animals is routinised, systemic, and legitimised. He conceptualises this as a ‘*war against animals*’, whereby humans assume rights of domination and entitlement to animal bodies. According to the author, CAS allows researchers to also be activists, describing links with ecofeminism, as both are engaged with the exclusion of women, racialised, the poor, and non-human animals. The author calls for further engagement with contemporary feminist scholarship, as masculinity drives practices, institutions, and geopolitical decision-making, while feminine qualities of care, compassion, and empathy towards non-humans are marginalised. Simultaneously, the author discusses the need to move away from the anthropocentric focus of climate justice which currently largely ignores the systemic injustice to animals, while animal ethics are largely reduced to ‘welfare’ rather than including animals in bio- and climate ethics.

Chapter three focuses on animal suffering and extinction as a result of climate change, highlighting how the climate and biodiversity crisis are caused by the same capitalist development that assumes human sovereignty over non-human animals. The chapter discusses a wide range of impacts of the climate crisis on animals, through a variety of causes and cascading pathways, arguing that climate change is a ‘*question of animal ethics, human-animal relations*’ that ‘*cannot be addressed on the bases of (…) anthropocentric notions of justice alone*’. This is reflected in current policies and research reports by institutions supported by meat producers. The author describes how non-human species exploitation is inherent to our current economic model, resulting in individual animal suffering at scale.

Part II shifts focus to introduce potential transitions from existing meat cultures to veganism. In chapter five the author draws interesting parallels with childhood studies, highlighting how children, like animals, are seen as ‘*good and innocent*’, and remain excluded from political decisions around the animal-industrial complex and climate change, even if they are most affected. The author provides examples how children can make more informed choices, leading to a ‘*vegan educational culture*’. Through the introduction of the ‘*practice theory*’, the author aims to eliminate the dichotomy of individual versus structural change to address the status-quo of the animal-industrial complex. This is a useful contribution to the wider discourse on ‘*attitude, behaviour*’, and ‘*choice*’, as – rather than based on individual decisions – dietary choices can be seen as ‘*compound practices*’, related to the overall policy and practice context, based on ‘*competences, materials and meanings (such as masculinity)*’, reinforcing each other. The author lists some of the myths around meat-based diets for health, and specifically the Eurocentric ‘*nutrition transition*’ which is exported to the global South, rather than demand driven, with dietary changes resulting in increased burdens of disease instead.

The final three chapters describe three scenarios to a meat-free diet, starting with the shift to plant-based diets, which the author rejects as anthropocentric, to ‘*intersectional veganism*’, involving transformative social change through considering the interconnections of animal exploitation and capitalism, colonialism, social class inequalities, and cultural differences, and a reform of the food system towards multispecies flourishing rather than profit. The author criticises animal product replacements and ‘*techno-optimism*’ such as lab-grown meat, as merely ‘*cleansing*’ meat production of its ‘*violence against animals, ecosystems and human labour*’. Meanwhile, he considers these substitutes as poorly equipped to counter climate change and the biodiversity crisis. He further critiques the false dichotomy between the political and economic spheres, considering the obstacles for transition both political and economic, related to protectionism, profitability, short-term political and corporate interests.

While the book achieves its objective to some extent, its message on the importance of the inclusion of non-human animals into a transformation for climate change mitigation is not always clear among the extensive deliberations of theories and frameworks leading to veganism as the author’s contribution to changing human-animal relations. Through its descriptions of the links between CAS, the Capitalocene, pedagogy, and practice theory, the book arguably achieves more than its stated objective and, as a result, sometimes the narrative loses focus. In my view, the book could have been more concise, with a stronger focus on the descriptions of the relations and processes for transformation.

The main weakness of the book however is that at times it seems to reinforce the anthropocentrism it claims to reject. Early on, the author makes a great argument that engagement with the more-than human and contesting human-animal relations based on interspecies’ inequalities, consumerism, and economic growth, are essential to our response to the climate crisis. The author highlights however, that human health and environmental considerations currently remain a stronger factor in veganism than do ethics. The author notes a strong resistance to animal ethics messaging, and while animal welfare is once mentioned as a ‘*worthwhile goal in its own right*’, the author does not return to this statement, instead mentioning that we ‘*need to think carefully how ethical meanings are conveyed*’. Only at the very end is the capitalist food system targeted as the cause for much animal and planetary suffering, which – if mentioned earlier on – would have been a great segway between Part I and II.

The strongest point the book makes does therefore not necessarily relate to human-animal relations, but rather the need for a more critical stance towards capitalism and its negative impact on democracy, as we currently see being played out in real life. The author calls for allied communities of practice, including vegans, feminists, and climate activists, targeting avenues for transformative change through progressive politics. Concluding to have provided a ‘*major opportunity for international policymakers*’ to address the climate crisis through ‘t*ransformative change through human-animal relations*’, the author argues that ‘*now is the time for post-capitalist narratives, questioning human-animal relations and systemic problems of the animal-industrial complex, whose growth is about power, conflicting with animal welfare*’. While the proposed intersectional veganism and vegan climate justice go some way towards providing tools to do so, what remains lacking is constructing these ideas in practical community-based action and platforms, acknowledging the deep bond that exists between many humans and their animals, and indigenous knowledge on more harmonious food system and biodiversity symbiosis.

To conclude, I found this a compelling read reframing the narrative related to causes and harmful impacts of the animal-human bond on some of the causes and impact of climate change. I would recommend the book to academic audiences looking for a more intersectional approach to addressing climate change related challenges, animal and human nutrition, health, and food systems, from which policy-makers may also benefit. It provides an important introduction to the potential uses of CAS, and perhaps most importantly, provides a strong argument for the inclusion of social sciences across disciplines. While many of its arguments could be of benefit a wider audience, it might be a challenging read to non-specialist audiences, as the author presumes some level of familiarity with many of the underlying debates and historical, political, and socio-economic factors leading to the climate crisis.

